# Quality evaluation of *Polygonatum sibiricum* slices from different regions based on appearance traits and multi-index metabolites combined with TOPSIS and gray relation analysis

**DOI:** 10.3389/fphar.2025.1547398

**Published:** 2025-05-13

**Authors:** Cheng Wang, Ju Ye, Sisi Jiang, Xuguang He, Min Ma, Li Yin

**Affiliations:** ^1^ School of Pharmacy, Qinghai Minzu University, Xining, Qinghai, China; ^2^ Northwest Institute of Plateau Biology, Chinese Academy of Sciences (CAS), Key Laboratory of Plant Resources of Qinghai-Tibet Plateau in Chemical Research, Xining, Qinghai, China; ^3^ Department of Education of Guizhou Province, Zunyi Medical and Pharmacy College, Zunyi, Guizhou, China

**Keywords:** *P. sibiricum* slices, appearance characteristics, indicator metabolites, weighted TOPSIS-GRA fusion model, quality evaluation

## Abstract

**Introduction:**

Traditional Chinese medicine quality control faces challenges, lacking multidimensionality and reliable quantitative evidence. Comprehensive evaluation models based on external characteristics and multiple indicator metabolites are the future research direction. This study focuses on *Polygonatum sibiricum* slices, aiming to establish a method for its quality evaluation.

**Methods:**

The appearance traits of *P. sibiricum* slices were quantified, and the contents of six functional metabolites were determined. With eight traits and six metabolite contents as variables, principal component analysis (PCA) and orthogonal partial least-squares discrimination analysis (OPLS-DA) were performed. A weighted TOPSIS-GRA fusion model was established by combining the technique for order preference by similarity to ideal solution (TOPSIS) and gray relation analysis (GRA).

**Results:**

The six metabolites showed good linear relationships (R^2^ > 0.9992) within their respective ranges, with an average recovery rate of 98.54% - 103.07% (relative standard deviation less than 1.64%). Precision, stability, and repeatability met the relevant standards. There were significant differences in traits and metabolite contents among slices from different habitats. OPLS-DA identified differential quality-affecting markers. PCA showed that the first three principal components contributed over 80% of the cumulative variance, and 16 batches of slices were clustered into three categories by origin. The weighted TOPSIS-GRA fusion model indicated significant quality differences among slices from different regions, consistent with PCA and OPLS-DA clustering results.

**Discussion:**

The established multi-index content determination method is accurate and reliable for detecting metabolites in *P. sibiricum* slices. The PCA, OPLS-DA, and weighted TOPSIS-GRA fusion models are scientifically reliable. The correlation between appearance traits and product quality can be used to evaluate *P. sibiricum* slices from different regions, which is of great significance for quality control and standardization of traditional Chinese medicine.

## 1 Introduction

Traditional Chinese medicine (TCM) is the foundation for innovative developments in the medical industry in China and abroad, and forms the material basis for ensuring the clinical safety of associated treatments. The quality standards of TCM are key to the high-quality development of the industry. However, limitations in research ideas and methods mean that current research on TCM quality control lacks multidimensionality, chain continuity, and integration. TCM evaluation methods are based on the concept of “identifying patterns and discussing quality” (Kang et al., 2024), which makes it difficult to assess the internal metabolites and their efficacy, and results in a lack of reliable quantitative evidence. Modern physical and chemical evaluation methods are based on quantifying the content of a single or group of metabolites. Although accurate quantification can be achieved, the results are difficult to relate to clinical efficacy and safety. Moreover, the inherent characteristics of TCM mean that quality cannot be judged solely by metabolites. Although the external characteristics of medicinal materials are closely related to their internal metabolites and efficacy, the correlation and internal mechanisms acting between numerous characteristics and complex metabolites present several research challenges. Therefore, comprehensive evaluation models based on external characteristics and multiple indicator metabolites have become the mainstream research direction for future TCM quality control.

The technique for order preference by similarity to ideal solution (TOPSIS) assigns weights to multiple indicators, using the degree of proximity to the idealized target as a benchmark for the comprehensive evaluation of samples. This eliminates subjective factors brought about by “identifying patterns and discussing quality”, and ensures objectivity, accuracy, and a scientific basis (Xu et al., 2024). Gray relation analysis (GRA) has been used to identify complex relationships between multiple indicators, and can analyze and compare the factors that have the greatest impact on samples based on the degree of interrelation among multiple factors. In this way, GRA intuitively reflects the comprehensive value of each indicator (Chen et al., 2018).

*Polygonatum sibiricum*, a historically revered tonic botanical drug, is traditionally used to nourish vital energy (Qi), dispel rheumatism, and harmonize visceral functions. Modern pharmacological studies reveal its rich metabolites of polysaccharides, saponins, flavonoids, alkaloids, and phenylpropanoids, which collectively contribute to hypoglycemic, lipid-lowering, antioxidant, anti-aging, and antitumor activities (Yang et al., 2021; Zhang et al., 2024a). Despite polysaccharides being recognized as primary active metabolites in *P. sibiricum* (Chinese Pharmacopoeia, 2020), emerging evidence underscores the synergistic roles of secondary metabolites in its therapeutic efficacy. To address regional quality disparities and align with the “Quality Marker (Q-Marker)” concept (Yuan and Wang, 2024), this study integrates morphological traits (long diameter, short diameter, single weight, thickness, chromaticity values) with quantification of five bioactive metabolites: baicalein, liquiritigenin, neoliquiritin, 3′-methoxydaidzin, and diosgenin. The selection of baicalein, liquiritigenin, neoliquiritin, 3′-methoxydaidzin, and diosgenin was driven by a hierarchical rationale rooted in pharmacological relevance, alignment with TCM synergy principles, and methodological rigor. First, the pharmacological significance of these metabolites directly mirrors *P*. *sibiricum*’s traditional and modern therapeutic applications. For instance, baicalein, a flavonoid, was prioritized for its NF-κB-mediated anti-inflammatory and antioxidant properties, which align with the botanical drug’s historical use in rheumatism management (Gao et al., 2024; Mabalirajan et al., 2013). Moreover, liquiritigenin and its glycoside neoliquiritin were included due to their immunomodulatory and neuroprotective effects, supporting the botanical drug’s role in Qi replenishment and immune enhancement (Yao et al., 2022; Xu et al., 2024). Furthermore, 3′-methoxydaidzin, an isoflavone with estrogenic activity, reflects *P. sibiricum*’s yin-nourishing applications in alleviating menopausal symptoms (Hou et al., 2024), while diosgenin, a steroidal saponin, was selected for its antitumor and lipid-lowering mechanisms via apoptosis induction and metabolic regulation (Xu et al., 2022; Zhang et al., 2021). Critically, these metabolites collectively embody the TCM paradigm of multi-metabolite synergy, wherein therapeutic efficacy arises from interconnected interactions rather than isolated constituents (Xiao et al., 2025). Finally, methodological coherence was ensured by integrating prior Q-Marker predictions (Jiang et al., 2017; Wang et al., 2024) with TOPSIS-GRA frameworks, enabling robust correlations between morphological traits and metabolite levels to harmonize traditional and modern quality standards.

By synthesizing morphological and multi-metabolite data, this work establishes a scientifically robust framework for evaluating *P. sibiricum* quality, offering insights for authenticity verification, origin tracing, and standardized industrial practices. The findings advance TCM quality standardization and underscore the necessity of integrating traditional wisdom with contemporary analytical methodologies.

## 2 Materials and methods

### 2.1 Test drugs

**FIGURE 1:**
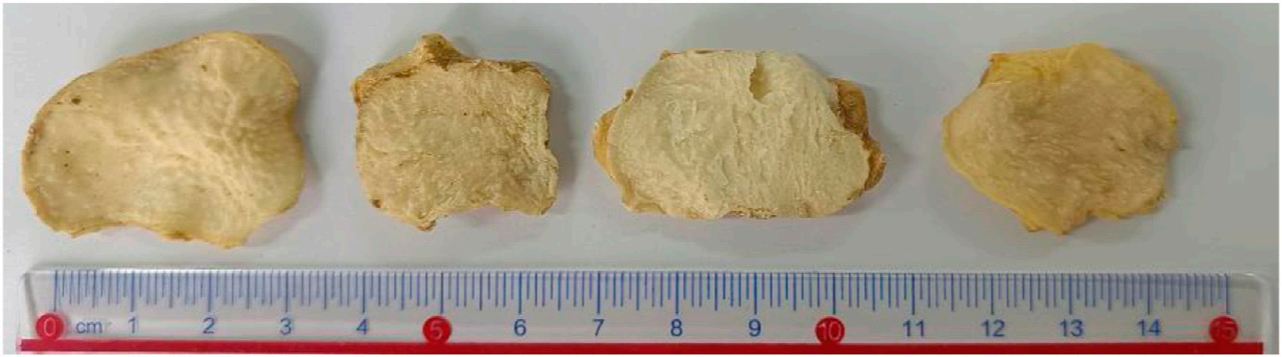
Typical photographs of sliced *P*. *sibiricum*

The reference substances of baicalein (batch No. A1119L021, purity 98.5%), liquiritigenin (batch No. A105J021, purity 98.5%), neoliquiritin (batch No. 2230816001, purity 98.5%), 3′-methoxydaidzin (batch No. Y14F10w79687, purity 98.5%), and diosgenin (batch No. A922E024, purity 98.5%) were procured from Beijing Solarbio Science & Technology Co., Ltd. High-performance liquid chromatography-grade acetonitrile, methanol, ethanol, and glacial acetic acid were supplied by Tianjin Fuyu Fine Chemical Co., Ltd. The P. sibiricum slices were authenticated as the dried rhizomes of Polygonatum by Professor Cairang Nanjia from the School of Pharmacy at Qinghai Minzu University. The *P. sibiricum* specimens used in this study are preserved in the Specimen Laboratory of the School of Pharmacy, Qinghai Minzu University. Table 1 details the information regarding the sample collection sites. As shown in Figure 1, the slices of *P*. *sibiricum* are presented, which can provide a visual reference for the morphological characteristics of this TCM.

**TABLE 1:** Origins of *P. sibiricum* slices.

No.	Source
S1	Tongnan, Chongqing
S2	Shizhu, Chongqing
S3	Changshou, Chongqing
S4	Hechuan, Chongqing
S5	Mianyang, Sichuan
S6	Luzhou, Sichuan
S7	Jianyang, Sichuan
S8	Yibin, Sichuan
S9	Emei, Sichuan
S10	Zhongjiang, Sichuan
S11	Anshun, Guizhou
S12	Kaili, Guizhou
S13	Bijie, Guizhou
S14	Zunyi, Guizhou
S15	Jindong, Yunnan
S16	Jinggu, Yunnan

### 2.2 Instruments

To prepare, examine, and characterize the samples, we used a Shimadzu LC-20A High Performance Liquid Chromatograph (Shimadzu, Japan); Analytical High-Performance Liquid Chromatography Column (Hypersil ODS-C18: Elite Suzhou Analytical Instruments Co., Ltd., China); KQ-50DA CNC Ultrasonic Cleaner (Kunshan Hechuang Ultrasonic Instrument Co., Ltd., China); XS105DU Electronic Balance (Shanghai Mettler Toledo Co., Ltd., China); SHE-D Vacuum Pump (Gongyi Yuhua Instrument Co., Ltd., China); UPH-IV Ultra Pure Water Machine (Chengdu Ultra Pure Science & Technology Co., Ltd., China); and 3nh Color Difference Meter (Guangdong Sannshi Intelligent Technology Co., Ltd., China).

### 2.3 Appearance characteristic measurement

Slices of *P*. s*ibiricum* from diverse origins were thoroughly homogenized and arranged to form a square configuration. Subsequently, a partition board was employed to divide the square into four congruent sections along the diagonal. Two diagonally opposed sections were selectively retained, while the remaining two sections were discarded. The two selected portions were then re-combined, and this procedure was iteratively repeated until the number of samples per batch attained 20. A precision balance and a vernier caliper were utilized to measure the mass, thickness, major diameter, and minor diameter of the samples. Subsequently, the average values of these measured parameters were calculated.

### 2.4 Decoction pieces chromaticity measurement

A colorimeter was employed to conduct measurements under the D65 light source, with a measurement wavelength range spanning from 180 to 740 nm and a measurement field of 10°. Prior to sample measurement, black and white calibration was performed on the colorimeter. Each batch, consisting of 20 samples, was placed within the test chamber, ensuring that the colorimeter’s measuring spot was precisely aligned at the center of the test chamber. The colorimetric values *L* ^*^, *a* ^*^, and *b* ^*^ were then recorded. Here, *L* ^*^ denotes the brightness of the sample color, *a* ^*^ represents the red - green gradient axis, and *b* ^*^ represents the yellow - blue gradient axis, where larger absolute values correspond to more intense colors (Yang et al., 2020). Each measurement was replicated three times, and the average values were calculated. Subsequently, the comprehensive color difference value ∆*E* for each batch of samples was computed according to Equation 1.

$$∆E={\left(L^{*2}+a^{*2}+b^{*2}\right)}^{1/2}$$ (1)

### 2.5 Determination of indicator metabolite contents

#### 2.5.1 Preparation of standard solution

The requisite amounts of the reference substances, namely, baicalein, liquiritigenin, neoliquiritin, diosgenin, and 3′-methoxydaidzin, were accurately weighed. These substances were then dissolved in chromatographic-grade methanol and transferred to a 25 mL volumetric flask. The volume was adjusted to the mark with the same solvent, thereby preparing a mixed reference substance solution. The resulting mass concentrations of baicalein, liquiritigenin, neoliquiritin, diosgenin, and 3′-methoxydaidzin in the solution were 0.0816, 0.094, 0.0828, 0.098, and 0.0796 mg·mL^−1^, respectively.

#### 2.5.2 Preparation of test Sample solution

Appropriate quantities of *P. sibiricum* samples were meticulously pulverized and sieved. Subsequently, precisely 2.00 g of the resultant powder was weighed and transferred into a conical flask. To this, 40 mL of 70% ethanol was added, and the mixture was subjected to sonication for a duration of 30 min. After sonication, the mixture was cooled and then filtered. A portion of the filtrate was collected, and the above-mentioned procedures were reiterated for the filter residue. The filtrates were then pooled together and concentrated under reduced pressure to yield a paste. This paste was re-dissolved in methanol to a final volume of 10 mL. Subsequently, 100 µL of a 10 g·L^−1^ chitosan-glacial acetic acid solution was added dropwise, and the resulting solution was left to stand overnight. Finally, the solution was filtered through a 0.22 - µm filter membrane to obtain the test solution.

#### 2.5.3 High-performance liquid chromatography conditions

**FIGURE 2:**
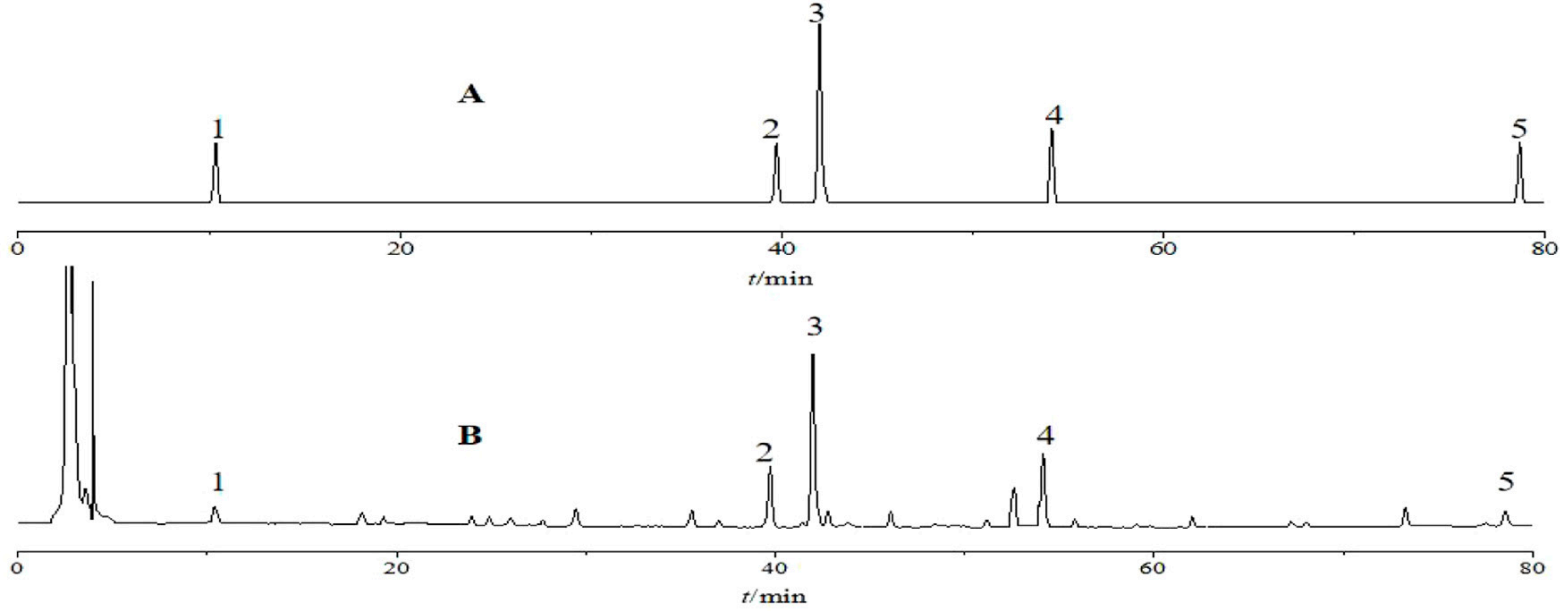
Chromatograms of standard solution **(A)** and sample solution **(B)** obtained by high-performance liquid chromatography. Note: 1-Neoliquiritin; 2-Liquiritigenin; 3–3′-Methoxydaidzin; 4-Baicalein; 5-Diosgenin.

High-performance liquid chromatography (HPLC) analysis was performed using a Hypersil ODS-C18 column (250 mm × 4.6 mm, 5 µm particle size). The detection wavelength was set at 230 nm. The mobile phase, consisting of acetonitrile (A) and water (B), was applied for gradient elution with the following program: 0–20 min, 5%–30% A; 20–30 min, 30%–40% A; 30–40 min, 40%–60% A; 40–80 min, 60%–100% A. The injection volume was 10 μL, the column temperature was maintained at 30°C, and the flow rate was 1.0 mL·min^−1^. Chromatograms of the reference and test samples of *P. sibiricum* slices under these conditions are shown in Figure 2.

#### 2.5.4 Investigation of the linear correlation

In accordance with the chromatographic conditions described in Section 2.5.3, injection volumes of 4, 8, 12, 16, 20, and 24 µL were prepared. The corresponding chromatographic peak areas were recorded. Linear regression equations were established, with the injection volume as the independent variable (X, abscissa) and the peak area as the dependent variable (Y, ordinate).

#### 2.5.5 Precision test

An appropriate volume of the mixed standard solution was subjected to six consecutive injections (10 μL per injection) under the chromatographic conditions specified in Section 2.5.3. The chromatographic profiles were recorded and the relative standard deviation (RSD) values of the peak areas for five target analytes - baicalein, liquiritigenin, neoliquiritin, diosgenin, and 3′-methoxydaidzein - were systematically calculated to evaluate the precision of the analytical method.

#### 2.5.6 Stability test

A sample (S1) solution of P. sibiricum slices was extracted and detection was performed every 4 h over a 24-h period. The baicalein, liquiritigenin, neoliquiritin, diosgenin, and 3′-methoxydaidzin peak areas were recorded.

#### 2.5.7 Repeatability test

Six samples of *P. sibiricum* powder (S1) were prepared in parallel according to the method described in Section 2.5.2. The samples were injected according to the chromatographic conditions detailed in Section 2.5.3, and the peak area was determined.

#### 2.5.8 Sample recovery test

Nine aliquots of S1 powder containing known amounts of the five target analytes (baicalein, liquiritigenin, neoliquiritin, diosgenin, and 3′-methoxydaidzin) were prepared. Three aliquots each of low-, medium-, and high-mass-fraction control solutions (three replicates per concentration level) were then spiked. The test solution was prepared according to the method in Section 2.5.2, and chromatographic analysis was performed under the conditions described in Section 2.5.3. Finally, the average recoveries and RSD were calculated by comparing the measured amounts with the spiked amounts.

#### 2.5.9 Content determination

Sixteen batches of samples were pulverized and sieved, and then test solutions were prepared according to the method detailed in Section 2.5.2, after which the prepared test solutions were diluted with methanol at a ratio of 1:10, vortex-mixed for 30 s, and allowed to stand for 5 min.The prepared samples were injected under the chromatographic conditions specified in Section 2.5.3, and the corresponding peak areas were recorded. The contents of baicalein, liquiritigenin, neoliquiritin, diosgenin, and 3′-methoxydaidzin were calculated by applying the pre-established linear regression equations.

### 2.6 Determination of polysaccharide content in *P. sibiricum* slices

#### 2.6.1 Drawing of standard curve

The anhydrous glucose was dried to constant weight at 105°C, and precisely 33 mg was taken and added to a 100-mL volumetric flask. Then, 0, 0.2, 0.5, 1, 1.5, and 2 mL of glucose solution was added to six 10-mL test tubes, with distilled water added up to 2 mL where necessary, followed by the addition of 0.2% anthrone–sulphuric acid solution in an ice-water bath up to 10 mL. The test tubes were left at room temperature before being subjected to a boiled water bath for 10 min, followed by a room temperature water bath for 10 min. The absorbance was then measured at 620 nm. The standard curve was plotted, with the concentration c of glucose standard solution as the horizontal coordinate and the absorbance A as the vertical coordinate. The results showed that there was a good linear relationship between absorbance and concentration, with the regression equation A = 34.661c+0.8079 achieving *R* ^2^ = 0.9998.

#### 2.6.2 Determination of polysaccharide content

The sample powder was dried at 60°C to constant weight. Subsequently, 0.25 g of the dried powder was accurately weighed and transferred into a round-bottomed flask. Then, 150 mL of 80% ethanol was added. Ultrasonic extraction was performed for 15 min, followed by reflux extraction in a boiling water bath for 1 h. The hot solution was filtered, and the residue was washed three times with 10 mL of 80% ethanol each. The residue and filter paper were transferred to another flask, and 150 mL of distilled water was added. Reflux extraction in a boiling water bath was repeated for 1 h, followed by hot filtration. The filtrate and washings were combined and further washed four times with 10 mL of distilled water each (filtered while hot). The residue and flask were also washed four times with 10 mL of hot distilled water. All filtrates and wash solutions were combined, cooled to room temperature, and transferred quantitatively to a 250 mL volumetric flask. The volume was adjusted to the mark with distilled water, and the flask was shaken thoroughly to obtain the test solution.

Exactly 1 mL of the test solution was pipetted into a 10 mL stoppered test tube, and 2 mL of distilled water was added. After thorough mixing, 0.2% anthrone-sulfuric acid solution was slowly added to the marked volume in an ice-water bath. The mixture was shaken gently, cooled, and then placed in a boiling water bath for 10 min. After cooling in a room-temperature water bath for 10 min, the solution was equilibrated at ambient conditions. Using distilled water as the blank, absorbance was measured at 620 nm. The absorbance value was substituted into the pre-established linear regression equation to calculate the polysaccharide content.

### 2.7 Statistical analysis

In the SIMCA 14.1 software, the orthogonal partial least-squares discrimination analysis (OPLS-DA) function was used to analyze the variable importance in the projection (VIP) of the indicator metabolites and polysaccharides. Duncan’s multiple range test (*P* < 0.05) was applied to determine the level of statistical significance after principal component analysis (PCA) and one-way analysis of variance in IBM’s SPSS Statistics 27.0 software. All data (mean ± standard deviation) were determined independently in triplicate.

### 2.8 Weighted TOPSIS-GRA fusion modeling

#### 2.8.1 Weighted TOPSIS data normalization

The analysis described in the previous subsections suggests that larger values of the mass, thickness, long diameter, short diameter, baicalein, liquiritigenin, neoliquiritin, diosgenin, 3′-methoxydaidzin, and polysaccharides of *P. sibiricum* slices are preferable. These variables were normalized using Equation 2. In contrast, smaller values of *L* ^*^, *a* ^*^, *b* ^*^, and *∆E* are desirable. These variables were normalized using Equation 3 (Liao et al., 2024).

$$Y_{\text{ij}}=\frac{X_{\text{ij}}-\min\left(x_{j}\right)}{\max\left(x_{j}\right)-\min\left(x_{j}\right)}$$ (2)

$$Y_{\text{ij}}=\frac{\max\left(x_{j}\right)-X_{\text{ij}}}{\max\left(x_{j}\right)-\min\left(x_{j}\right)}$$ (3)

Note: $Y_{\text{ij}}$ is the normalization result and $X_{\text{ij}}$ is the value of each indicator measurement.

#### 2.8.2 Weighted TOPSIS Calculation for each evaluation indicator

The variable importance in projection (VIP) values of the 14 indicators derived from orthogonal partial least-squares discriminant analysis (OPLS-DA) were assigned as the weighting coefficients (Q_j_). The weighting matrix values (Z_ij_) for each indicator were then computed using Equation 4. Based on this matrix, the positive and negative ideal samples (Z^+^) and (Z^−^) were defined. Finally, the distances from each evaluation indicator to the ideal samples (d_i_^+^) and (d_i_^−^) were calculated via Equations 5, 6 (Zheng et al., 2024).

$$Z_{\text{ij}}=Y_{\text{ij}}\times Q_{j}$$ (4)

$${d_{i}}^{+}=\sqrt{\sum_{j=1}^{n}{\left(Z_{ij}-{Z_{j}}^{+}\right)}^{2}}$$ (5)

$${d_{i}}^{-}=\sqrt{\sum_{j=1}^{n}{\left(Z_{ij}-{Z_{j}}^{-}\right)}^{2}}$$ (6)

#### 2.8.3 GRA standardization

Because of the significant differences in the results of the 14 indicators measured in the 16 batches of *P. sibiricum* slices, data standardization was required prior to GRA (Li et al., 2024). The data were standardized using Equation 7.

$$M_{ij}=X_{ij}/X_{mj}$$ (7)

Note: $M_{ij}$ is the result of the standardization process for each indicator and $X_{mj}$ is the average of the results for each indicator.

#### 2.8.4 Calculation of GRA correlation coefficients

The GRA method evaluates the interrelationships among research objects by quantifying their degrees of relevance. Higher GRA correlation values indicate stronger associations and superior quality of the objects (Fu et al., 2019). Among the 16 batches of *P. sibiricum* slices, parameters including mass, thickness, long diameter, short diameter, and the contents of baicalein, liquiritigenin, neoliquiritin, diosgenin, 3′-methoxydaidzin, and polysaccharides were classified as positive indicators. For these positive indicators, the maximum values observed across all batches were selected as the optimal reference sequence, while the minimum values were designated as the worst reference sequence during GRA standardization. Conversely, the color parameters *L* ^*^, *a* ^*^, *b* ^*^, and ∆*E* were considered negative indicators. For these, the maximum values from GRA standardization were assigned as the worst reference sequence, and the minimum values as the optimal reference sequence. The correlation coefficients between each evaluation index and both reference sequences (optimal and worst) were calculated using Equations 8, 9.

$$E_{jb}^{i}=\frac{{∆}_{\min}+\alpha {∆}_{\max}}{\left|M_{ij}-M_{bj}\right|+\alpha {∆}_{\max}}$$ (8)

$$E_{js}^{i}=\frac{{∆}_{\min}^{\prime }+\alpha {∆}_{\max}^{\prime }}{\left|M_{ij}-M_{sj}\right|+\alpha {∆}_{\max}^{\prime }}$$ (9)

Note: $E_{jb}^{i},E_{js}^{i}$ are the correlation coefficients of the best and worst reference sequences; $M_{bj},M_{sj}$ are the best and worst reference sequences; ${∆}_{\min}=\min\left|M_{ij}-M_{bj}\right|$ , ${∆}_{\max}=\max\left|M_{ij}-M_{bj}\right|$ , ${∆}_{\min}^{\prime }=\min\left|M_{ij}-M_{sj}\right|$ , ${∆}_{\max}^{\prime }=\max\left|M_{ij}-M_{sj}\right|$ ; α is the resolution coefficient, which takes a value of 0.5 (Zhang et al., 2020).

#### 2.8.5 Calculation of relative correlation degree of GRA

Using the process explained in Section 2.8.4, the relative correlation of each batch was calculated. The relative correlation is the mean value of the correlation coefficients of all evaluation indicators of the research object. Thus, the correlation of *P. sibiricum* slices with respect to the optimal and the worst reference sequences was calculated using Equations 10, 11 (Qu et al., 2019), respectively.

$${q_{i}}^{+}=\frac{1}{14}\sum_{j=1}^{14}E_{jb}^{i}$$ (10)

$${q_{i}}^{-}=\frac{1}{14}\sum_{j=1}^{14}E_{js}^{i}$$ (11)

#### 2.8.6 Weighted TOPSIS-GRA fusion Modeling

Using Equations 12–15, the Euclidean distance and relative correlation of the reference sequence for 16 batches of *P. sibiricum* slices were subjected to dimensionless processing. Subsequently, post-processing by the weighted TOPSIS-GRA fusion model was applied using Equations 16, 17. *D*_i_^+^ and *Q*_i_^−^ denote the extent to which samples deviate from the ideal sample; smaller values indicate closer proximity to the ideal sample. Conversely, *D*_i_^−^ and *Q*_i_^+^ signify the extent to which samples approach the ideal sample; larger values suggest a closer resemblance to the ideal sample. *G*_i_^+^ offers a comprehensive assessment of how closely all samples approximate the ideal sample; higher values equate to superior sample quality. *G*_i_^−^ provides a comprehensive evaluation of the distance of all samples from the ideal sample; higher values correspond to lower sample quality. The relative closeness κ was determined using Equation 18; higher values signify higher comprehensive sample quality.

$${D_{i}}^{+}={d_{i}}^{+}/\max\;{d_{i}}^{+}$$ (12)

$${D_{i}}^{-}={d_{i}}^{-}/\max\;{d_{i}}^{-}$$ (13)

$${{\;Q}_{i}}^{+}={q_{i}}^{+}/\max\;{q_{i}}^{+}$$ (14)

$${Q_{i}}^{-}={q_{i}}^{-}/\max\;{q_{i}}^{-}$$ (15)

$${G_{i}}^{+}=\alpha {D_{i}}^{-}+\beta {Q_{i}}^{+}$$ (16)

$${G_{i}}^{-}=\alpha {D_{i}}^{+}+\beta {Q_{i}}^{-}$$ (17)

$$\kappa ={G_{i}}^{+}/\left({G_{i}}^{+}+{G_{i}}^{-}\right)$$ (18)

Note: *α* and *β* are correlation coefficients, both taking a value of 0.5 (Yan et al., 2024).

## 3 Results

### 3.1 Appearance characteristics of *P. sibiricum* slices

The measurements of appearance-characteristic indices are presented in Table 2. As shown in the table, the 16 batches of samples exhibit significant differences among different origins based on eight evaluated traits.

**TABLE 2:** Appearance traits of samples of *P. sibiricum* slices from different producing areas (*x ± s*, *n* = 3).

No.	Mass/g	Thickness/mm	Long diameter/mm	Short diameter/mm	Chromaticity values			
					*L* ^*^	*a* ^*^	*b* ^*^	*∆E*
S1	4.028 ± 2.251c	2.104 ± 1.501de	45.818 ± 3.601c	17.590 ± 2.021cde	72.468 ± 0.020f	2.302 ± 0.005f	29.906 ± 0.016ab	78.430 ± 0.051 d
S2	7.816 ± 3.052a	3.188 ± 1.103abc	78.940 ± 2.835a	20.734 ± 0.857b	74.332 ± 0.016ef	3.064 ± 0.030de	10.752 ± 0.007f	75.168 ± 0.652e
S3	2.248 ± 2.057g	2.046 ± 1.106de	41.302 ± 3.006de	19.118 ± 1.503bc	75.362 ± 0.059cdef	3.628 ± 0.018bcd	27.628 ± 0.020bcd	80.349 ± 0.509bcd
S4	3.400 ± 2.967 d	2.556 ± 1.214bcd	39.528 ± 2.809ef	16.886 ± 2.557cdef	80.412 ± 0.036a	1.130 ± 0.029g	24.986 ± 0.021de	84.212 ± 0.209a
S5	3.464 ± 2.908cd	1.948 ± 0.869def	41.820 ± 3.290de	23.918 ± 1.836a	74.456 ± 0.059ef	2.770 ± 0.010eff	30.444 ± 0.069ab	80.487 ± 0.093bcd
S6	3.235 ± 3.907de	1.890 ± 0.680def	42.380 ± 3.309 d	17.068 ± 2.083cdef	76.596 ± 0.068bcde	5.588 ± 0.009a	28.158 ± 0.019abc	81.799 ± 0.092abc
S7	3.406 ± 1.208 d	3.206 ± 0.830abc	45.570 ± 2.075c	15.944 ± 1.153def	74.366 ± 0.016ef	2.658 ± 0.035ef	29.074 ± 0.082ab	79.892 ± 0.021cd
S8	7.846 ± 4.085a	3.280 ± 1.410ab	78.558 ± 4.510a	20.882 ± 2.59b	74.262 ± 0.005ef	3.106 ± 0.027de	12.384 ± 0.017f	75.351 ± 0.013e
S9	2.726 ± 1.570efg	1.378 ± 0.570ef	43.800 ± 3.917cd	19.078 ± 2.907bc	75.694 ± 0.019bcde	3.754 ± 0.076bc	30.940 ± 0.038a	81.859 ± 0.043abc
S10	2.388 ± 2.037fg	1.262 ± 0.029f	42.556 ± 4.162 d	19.780 ± 1.277bc	75.266 ± 0.030cdef	3.932 ± 0.020b	29.036 ± 0.065ab	80.768 ± 0.045bcd
S11	1.540 ± 0.970h	1.792 ± 0.561def	36.940 ± 3.009fg	15.680 ± 2.309ef	78.168 ± 0.089abc	1.056 ± 0.029g	24.768 ± 0.057de	82.005 ± 0.065abc
S12	2.496 ± 1.095fg	1.898 ± 0.751def	53.348 ± 4.068b	18.264 ± 3.005bcde	78.520 ± 0.037ab	−0.606 ± 0.015h	24.046 ± 0.025e	82.122 ± 0.028abc
S13	2.218 ± 1.305g	2.082 ± 1.073de	34.414 ± 3.507g	14.454 ± 2.801f	77.798 ± 0.034abcd	2.710 ± 0.011ef	25.820 ± 0.079cde	82.015 ± 0.044abc
S14	7.214 ± 1.808b	3.272 ± 1.283ab	78.974 ± 5.207a	20.804 ± 3.051b	74.130 ± 0.022ef	3.068 ± 0.073de	11.882 ± 0.035f	75.139 ± 0.053e
S15	2.896 ± 1.099def	2.432 ± 1.677cd	43.618 ± 2.033cd	18.728 ± 2.006bcd	77.452 ± 0.031bcd	4.044 ± 0.016b	28.652 ± 0.039abc	82.681 ± 0.025ab
S16	7.478 ± 2.331ab	3.950 ± 1.922a	78.460 ± 3.367a	20.938 ± 3.303b	74.920 ± 0.088def	3.170 ± 0.075cde	11.446 ± 0.076f	75.856 ± 0.077e

Note: Different letters in the same column indicate *P* < 0.05.

### 3.2 Methodological review

#### 3.2.1 Linear range Inspection

The standard curve of five quality metabolites was plotted, and the linear regression equation, correlation coefficient, and linear range of each reference substance was calculated. The results are presented in Table 3.

**TABLE 3:** Linear relationships for the five metabolites.

Metabolite	Linear equation	R^2^	Linear range/µg
Baicalein	Y = 8872.4X-53014	0.9992	0.9792∼2.6112
Liquiritigenin	Y = 14645X-14574	0.9996	0.3760∼2.2560
Neoliquiritin	Y = 189982X-49663	0.9994	0.3312∼1.9872
Diosgenin	Y = 341895X-216226	0.9993	0.3920∼2.3520
3′-Methoxydaidzin	Y = 14128X-22450	0.9995	0.3184∼1.9104

#### 3.2.2 Precision test results

The RSD values for baicalein, liquiritigenin, neoliquiritin, diosgenin, and 3′-methoxydaidzin were determined to be 1.75%, 0.65%, 0.31%, 1.56%, and 1.06%, respectively. These findings indicate that the precision of the analytical instrument meets the requirements of the test.

#### 3.2.3 Stability test results

The RSD values of baicalein, liquiritigenin, neoliquiritin, diosgenin, and 3′-methoxydaidzin were found to be 1.78%, 1.71%, 1.96%, 1.10%, and 1.98%, respectively. These results show that the solutions of P. sibiricum slices attain good stability within 24 h.

#### 3.2.4 Repeatability test results

The RSD values of baicalein, liquiritigenin, neoliquiritin, diosgenin, and 3′-methoxydaidzin were found to be 1.90%, 1.86%, 1.96%, 1.66%, and 0.92%, respectively. These results indicate that the method has good repeatability.

#### 3.2.5 Sample recovery test results

The baicalein, liquiritigenin, neoliquiritin, diosgenin, and 3′-methoxydaidzin average recoveries were 99.17%, 100.54%, 98.54%, 103.07%, and 99.08%; the RSD values were 1.51%, 0.87%, 1.64%, 1.09%, and 1.62%, respectively. These results demonstrate that the method satisfies the requirements of the spiked sample recovery test, indicating a high degree of accuracy.

### 3.3 Determination of indicator metabolites and polysaccharide content

The contents of the five indicator metabolites in *P. sibiricum* slices were quantified using the method detailed in Section 2.5. The polysaccharide content was assayed by the technique described in Section 2.6. The resultant data are presented in Table 4.

**TABLE 4:** Determination results of five metabolites and polysaccharide in *P. sibiricum* slices from different origins and batches (*n* = 3).

No.	Mass fraction/mg·g^−1^					
	Baicalein	Liquiritigenin	Neoliquiritin	Diosgenin	3′-methoxydaidzin	Polysaccharide
S1	37.286f	10.457bc	2.091cde	3.899a	8.690fg	186.3c
S2	68.295a	14.871a	2.339abc	3.894a	26.089b	194.0b
S3	54.156c	9.208d	2.242abcd	3.96a	9.911de	159.6e
S4	33.917g	11.058b	1.629e	3.919a	7.916gh	118.0g
S5	39.032e	7.937e	2.479abc	4.010a	8.725fg	172.7d
S6	42.454d	13.87a	2.390abc	3.902a	11.6934c	116.2f
S7	38.501e	10.796bc	2.516abc	3.890a	9.638def	184.0c
S8	68.648a	14.888a	2.727ab	3.869a	26.577b	209.5a
S9	55.529b	10.368bc	2.140cde	4.400a	10.105d	157.3e
S10	54.805bc	11.143b	2.218abcd	4.013a	9.431ef	156.1e
S11	30.177i	11.226b	1.637e	3.827a	7.464h	117.4g
S12	34.609g	11.268b	1.705de	3.814a	8.950ef	115.0g
S13	32.508h	14.527a	2.001cde	3.970a	8.828fg	114.8g
S14	68.634a	14.387a	2.463abc	4.338a	46.944a	196.1b
S15	55.345b	9.852cd	2.158bcde	3.912a	9.349ef	154.2e
S16	68.003a	14.324a	2.781a	4.429a	26.71b	181.0c

Note: Different letters in the same column indicate *P* < 0.05.

The results in Table 4 indicate that *P. sibiricum* slices of different origins exhibit significant differences in all indicator metabolites, except for diosgenin. The highest contents of baicalein, liquiritigenin, neoliquiritin, 3′-methoxydaidzin, and polysaccharides originated from samples S16, S5, S16, S6, and S8, respectively.

### 3.4 PCA and OPLS-DA

#### 3.4.1 PCA

PCA was applied to the 16 batches of *P. sibiricum* slices, with the appearance index data (mass, thickness, long diameter, short diameter, and chromaticity values *L* ^*^, *a* ^*^, *b* ^*^, *∆E*) and the contents of baicalein, liquiritigenin, neoliquiritin, diosgenin, 3′-methoxydaidzin, and polysaccharides as the variables. The analysis was performed using SPSS27.0 software. The results are presented in Tables 5, 6.

**TABLE 5:** PCA results.

Principal component	Initial eigenvalue			Extract the Sum of squares load		
	Summation	Variance contribution/%	Cumulative contribution/%	Summation	Variance contribution/%	Cumulative contribution/%
1	8.011	52.219	52.219	8.011	52.219	52.219
2	2.324	16.601	73.820	2.324	16.601	73.820
3	1.100	7.858	81.678	1.100	7.858	81.678
4	0.897	6.409	88.087			
5	0.762	5.439	93.526			
6	0.415	2.967	96.493			
7	0.225	1.607	98.101			
8	0.120	0.855	98.955			
9	0.063	0.453	99.408			
10	0.045	0.318	99.726			
11	0.032	0.227	99.954			
12	0.004	0.031	99.985			
13	0.002	0.014	99.999			
14	9.597 × 10^−5^	0.001	100.000			

**TABLE 6:** Composition matrix of 14 indicators in *P. sibiricum* slices.

Principal component	Payloads													
	Mass	Thickness	Long diameter	Short diameter	*L* ^*^	*a* ^*^	*b* ^*^	*∆E*	Baicalein	Liquiritigenin	Neoliquiritin	Diosgenin	3′-methoxydaidzin	Polysaccharide
1	0.962	0.670	0.946	0.619	−0.646	0.288	−0.843	−0.955	0.866	0.601	0.789	0.146	0.878	0.843
2	0.190	0.363	0.218	−0.365	0.623	−0.587	−0.490	0.040	−0.163	0.493	−0.411	0.619	0.208	−0.371
3	−0.044	−0.061	−0.059	−0.484	0.085	0.580	−0.031	0.038	0.104	0.575	0.131	−0.292	0.060	−0.251

**FIGURE 3:**
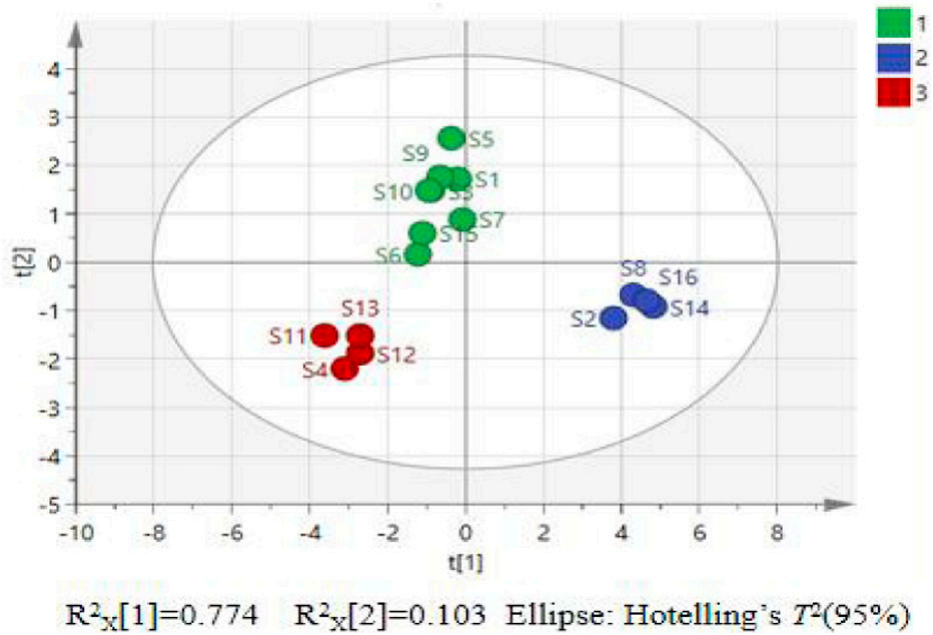
PCA score chart for 16 batches of *P. sibiricum* slices.

As shown in Table 5, the first three principal components exhibit eigenvalues greater than 1 (8.011, 2.324, and 1.100) (Zhang et al., 2024b), contributing variances of 52.219%, 16.601%, and 7.858%, respectively. Collectively, these components account for 81.678% of the total variation in *P. sibiricum* slices. Table 6 demonstrates that mass, thickness, long diameter, short diameter, *L* ^*^, *b* ^*^, *∆E*, baicalein, liquiritigenin, neoliquiritin, 3′-methoxydaidzin, and polysaccharides exhibit higher loadings on the first principal component, while *a* ^*^ and diosgenin are more strongly associated with the second principal component. A PCA model was constructed using SIMCA 14.1 software to explore the clustering patterns of *P. sibiricum* slices. The results are presented in Figure 3.

As shown in Figure 3, the 16 batches of *P. sibiricum* slices can be divided into three categories with significant clustering and dispersion, indicating that there are large differences in quality between the categories. Samples S4 and S11–S13 are clustered into one category; S2, S8, S14, and S16 are clustered into another category; and the remaining samples are clustered into a third category.

#### 3.4.2 OPLS-DA

**FIGURE 4:**
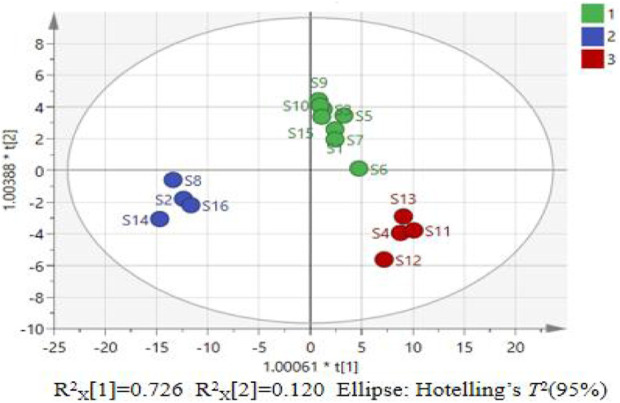
Score chart of OPLS-DA model for 16 batches of *P. sibiricum* slices.

**FIGURE 5:**
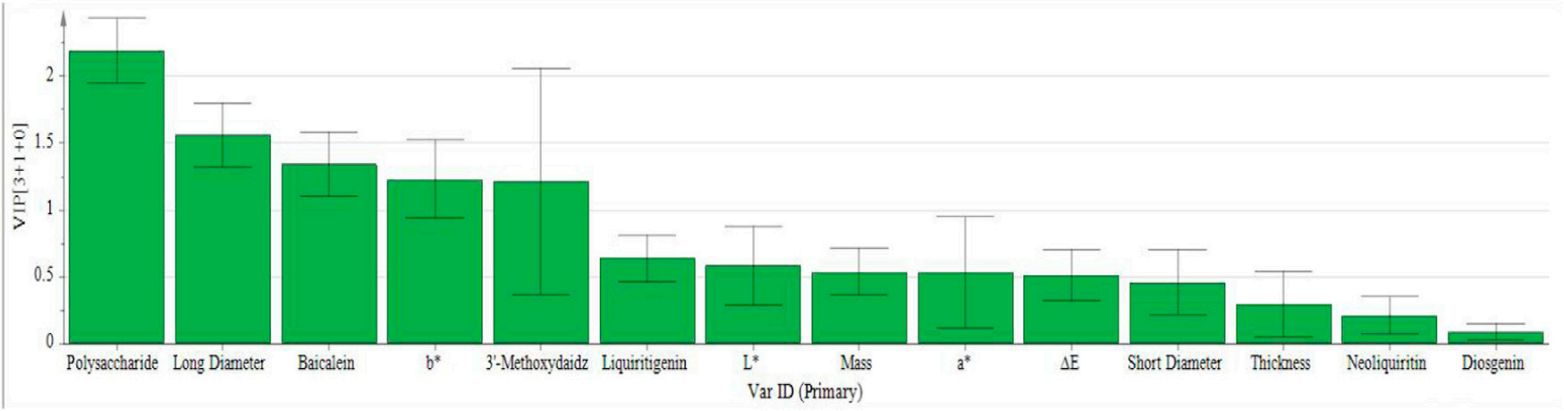
Variable importance in projection images of 16 batches of *P. sibiricum* slices.

To further investigate the factors influencing the quality differences between categories, the data matrix of 14 indicators in the 16 batches of *P. sibiricum* slices was imported into SIMCA 14.1 and OPLS-DA was applied. The results are presented in Figures 4, 5. The model parameters R^2^_X_ = 0.986, R^2^_Y_ = 0.971, and Q^2^ = 0.853 indicate that the model has good stability, reliability, and predictive ability.

**FIGURE 6:**
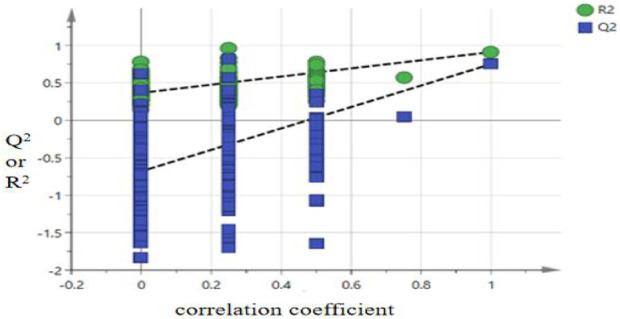
OPLS-DA replacement detection results.

Figure 4 reveals that the 16 batches of *P. sibiricum* slices are categorized into three distinct groups, consistent with the PCA results. Figure 5 presents the VIP values. Applying a VIP threshold >1 as the screening criterion (Gan et al., 2021), polysaccharides, long diameter, baicalein, *b* ^*^, and 3′-methoxydaidzin were identified as key markers for quality classification of *P. sibiricum* slices. These parameters exhibit substantial contributions to the overall quality evaluation and can effectively discern quality variations. The OPLS-DA model was validated through 200 permutation tests, with results displayed in Figure 6.

Figure 6 shows that the resultant R^2^ fitted curve intercepts the Y-axis below Y = 0.3 and the Q^2^ fitted curve intercepts the Y-axis below Y = 0. The results of cross validation analysis of variance (CV-ANOVA) indicated that the F value was 135.76 (*P* < 0.01), suggesting that the model was significantly effective. Therefore, the OPLS-DA model can be applied to evaluate the quality of *P*. *sibiricum* slices from different regions.

### 3.5 Matrix calculation of weighted TOPSIS

Table 7 presents the TOPSIS weighting matrix for each index, and Table 8 lists the distances between each evaluation index and the positive/negative ideal samples (*d*_i_^+^, *d*_i_^−^).

**TABLE 7:** Weighting matrix.

No.	*Z*_ij_													
	Mass	Thickness	Long diameter	Short diameter	*L* ^*^	*a* ^*^	*b* ^*^	*∆E*	Baicalein	Liquiritigenin	Neoliquiritin	Diosgenin	3′-methoxydaidzin	Polysaccharides
S1	0.2126	0.0938	0.3999	0.1529	0.5865	0.2839	0.0631	0.5147	0.2485	0.2322	0.0857	0.0123	0.0378	1.6544
S2	0.5363	0.2145	1.5615	0.3063	0.4489	0.2180	1.2320	0.3871	1.3325	0.6390	0.1317	0.0115	0.5735	1.8325
S3	0.0605	0.0873	0.2416	0.2274	0.3728	0.1693	0.2022	0.2439	0.8383	0.1172	0.1137	0.0211	0.0754	1.0367
S4	0.1590	0.1441	0.1794	0.1186	0.0000	0.3851	0.3633	0.0000	0.1307	0.2876	0.0000	0.0151	0.0139	0.0741
S5	0.1644	0.0764	0.2597	0.4615	0.4397	0.2435	0.0303	0.2913	0.3096	0.0000	0.1577	0.0283	0.0388	1.3397
S6	0.1449	0.0699	0.2794	0.1275	0.2817	0.0000	0.1698	0.1585	0.4291	0.5467	0.1412	0.0127	0.1376	0.0324
S7	0.1595	0.2165	0.3913	0.0726	0.4464	0.2531	0.1138	0.3746	0.2910	0.2634	0.1639	0.0110	0.0670	1.6011
S8	0.5389	0.2248	1.5483	0.3135	0.4541	0.2144	1.1324	0.4166	1.3449	0.6405	0.2037	0.0079	0.5885	2.1912
S9	0.1014	0.0129	0.3291	0.2255	0.3483	0.1584	0.0000	0.2819	0.8863	0.2240	0.0948	0.0846	0.0813	0.9834
S10	0.0725	0.0000	0.2855	0.2597	0.3799	0.1431	0.1162	0.2032	0.8610	0.2954	0.1093	0.0287	0.0605	0.9556
S11	0.0000	0.0590	0.0886	0.0598	0.1657	0.3915	0.3766	0.0468	0.0000	0.3031	0.0015	0.0019	0.0000	0.0603
S12	0.0817	0.0708	0.6640	0.1858	0.1397	0.5351	0.4207	0.1369	0.1549	0.3069	0.0141	0.0000	0.0457	0.0046
S13	0.0579	0.0913	0.0000	0.0000	0.1930	0.2486	0.3124	0.0460	0.0815	0.6073	0.0690	0.0225	0.0419	0.0000
S14	0.4849	0.2239	1.5628	0.3097	0.4638	0.2177	1.1630	0.4148	1.3443	0.5943	0.1547	0.0756	1.2157	1.8811
S15	0.2211	0.1303	0.3229	0.2084	0.2185	0.1334	0.1396	0.1568	0.8798	0.1765	0.0981	0.0141	0.0580	0.9118
S16	0.5074	0.2994	1.5448	0.3162	0.4054	0.2089	1.1896	0.3942	1.3223	0.5886	0.2137	0.0887	0.5926	1.5316

**TABLE 8:** Values of ${{\;d}_{i}}^{+}$ and ${d_{i}}^{-}$ in TOPSIS evaluation of *P. sibiricum* slices.

No.	${d_{i}}^{+}$	${d_{i}}^{-}$
S1	2.4680	1.9474
S2	0.8911	3.2666
S3	2.5621	1.4831
S4	3.3408	0.6916
S5	2.6619	1.6030
S6	3.1906	0.8812
S7	2.4437	1.8429
S8	0.7534	3.4573
S9	2.6008	1.4903
S10	2.5972	1.4601
S11	3.4302	0.6597
S12	3.1416	1.0532
S13	3.4690	0.7700
S14	0.5138	3.4376
S15	2.6030	1.4046
S16	1.0022	3.0753

The data presented in Tables 7,8 demonstrate that the distances between the 16 batches of samples and the positive ideal samples span from 0.5138 to 3.4690, while the distances to the negative ideal samples range from 0.6597 to 3.4573. Samples S2, S8, and S14 exhibit relatively higher quality, whereas S4, S11, and S13 show markedly lower quality. These findings indicate that *P. sibiricum* slices sourced from diverse geographical origins exhibit a heterogeneous quality profile, with statistically significant disparities.

### 3.6 Relative correlation degree of GRA

As shown in Table 9, the correlation coefficients of the 16 batches of *P. sibiricum* slices range from 0.3895 to 0.8094 for the optimal reference sequence and 0.3811 to 0.8085 for the worst reference sequence. Notably, samples S2, S8, S14, and S16 exhibit significantly stronger correlations with the optimal sequence (coefficients >0.7000), indicating superior quality. In contrast, samples S4, S11, S12, and S13 demonstrate higher correlations with the worst sequence (coefficients >0.7000), suggesting markedly inferior quality. These findings are consistent with the results of the weighted TOPSIS model.

**TABLE 9:** Values of ${q_{i}}^{+}$ and ${q_{i}}^{-}$ in GRA evaluation of *P. sibiricum* slices.

No.	${q_{i}}^{+}$	${q_{i}}^{-}$
S1	0.5191	0.5989
S2	0.7502	0.4266
S3	0.4470	0.6259
S4	0.4072	0.7625
S5	0.4988	0.6277
S6	0.4352	0.6847
S7	0.5157	0.5972
S8	0.7951	0.4186
S9	0.4783	0.6219
S10	0.4457	0.6405
S11	0.3895	0.8085
S12	0.4400	0.7084
S13	0.4219	0.7506
S14	0.8094	0.3811
S15	0.4346	0.6390
S16	0.7685	0.3872

### 3.7 Calculation of relative closeness κ

The results of the weighted TOPSIS-GRA fusion model demonstrate that the average relative closeness values of the 16 batches of *P. sibiricum* slices range from 0.2526 to 0.7635 (Table 10), indicating substantial quality variation across different geographical origins. The top four samples (S14, S8, S16, and S2) exhibit relative closeness values exceeding 0.7000, while the remaining samples fall below 0.6000. Notably, *P. sibiricum* slices from Zunyi (Guizhou Province), Yibin (Sichuan Province), Jinggu (Yunnan Province), and Shizhu (Chongqing Municipality) show significantly higher quality compared to other regions within the same provinces. These findings align with historical records of *P. sibiricum*’s traditional Dao-di producing areas (Ma et al., 2019), thereby validating the reliability of the TOPSIS-GRA fusion model.

**TABLE 10:** Quality sequencing of 16 batches of *P. sibiricum* slices.

No.	${D_{i}}^{+}$	${D_{i}}^{-}$	${{\;Q}_{i}}^{+}$	${Q_{i}}^{-}$	${G_{i}}^{+}$	${G_{i}}^{-}$	k	Sort
S1	0.7114	0.5633	0.6413	0.7408	0.6023	0.7261	0.4534	5
S2	0.2569	0.9448	0.9269	0.5276	0.9359	0.3923	0.7046	4
S3	0.7386	0.4290	0.5523	0.7741	0.4907	0.7564	0.3935	9
S4	0.9630	0.2000	0.5031	0.9431	0.3516	0.9531	0.2695	15
S5	0.7673	0.4637	0.6163	0.7764	0.5400	0.7719	0.4116	7
S6	0.9197	0.2549	0.5377	0.8469	0.3963	0.8833	0.3097	13
S7	0.7044	0.5330	0.6371	0.7387	0.5851	0.7216	0.4478	6
S8	0.2172	1.0000	0.9823	0.5177	0.9912	0.3675	0.7295	2
S9	0.7497	0.4311	0.5909	0.7692	0.5110	0.7595	0.4022	8
S10	0.7487	0.4223	0.5507	0.7922	0.4865	0.7705	0.3870	10
S11	0.9888	0.1908	0.4812	1.000	0.3360	0.9944	0.2526	16
S12	0.9056	0.3046	0.5436	0.8762	0.4241	0.8909	0.3225	12
S13	1.0000	0.2227	0.5213	0.9284	0.3720	0.9642	0.2784	14
S14	0.1481	0.9943	1.000	0.4714	0.9972	0.3089	0.7635	1
S15	0.7504	0.4063	0.5369	0.7904	0.4716	0.7704	0.3797	11
S16	0.2889	0.8895	0.9495	0.4789	0.9195	0.3839	0.7055	3

## 4 Discussion

### 4.1 Relationship between morphological traits and quality attributes in *P. sibiricum* slices

Morphological traits are critical yet undervalued in *P. sibiricum* quality assessment. Single-marker methods are insufficient; integrating morphological and metabolic perspectives is essential (Chinese Pharmacopoeia, 2020; Yuan and Wang, 2024). Processed *P. sibiricum* quality links to traits like slice long diameter and chromatic parameter *b* ^*^, reflecting regional environments and bioactive metabolite accumulation (Liang et al., 2025; Shao et al., 2025). Longer diameters indicate better-developed tissues (potentially higher active ingredients), while *b* ^*^ correlates with specific metabolites, signaling environmental impacts on biosynthesis. These traits are indispensable for quality evaluation, offering insights into metabolic processes and therapeutic efficacy.

### 4.2 Regional differences in polysaccharide and metabolite profiles of *P. sibiricum* slices

Geographical differences significantly shape *P. sibiricum* polysaccharide and metabolite profiles. OPLS-DA revealed distinct patterns: Sichuan and Guizhou samples exhibited elevated polysaccharides and region-specific metabolites (e.g., flavonoids, saponins) (Chen et al., 2024). Polysaccharides, a key pharmacopeial metabolite, underpin pharmacological effects, while metabolites like 3′-methoxydaidzin and baicalein—regionally variable—exhibit anti-inflammatory activities. Aligned with the “Dao-di botanical drugs” concept, these differences facilitate standardized sourcing and quality control in pharmaceutical applications.

### 4.3 Multi-model integrated approach for quality evaluation of *P. sibiricum* slices

This study introduces a TOPSIS-GRA methodology weighted by OPLS-DA-derived VIP values, providing a robust framework for *P. sibiricum* quality assessment. By incorporating VIP values—quantifying variable importance in sample differentiation—the model mitigates subjectivity, enabling objective, comprehensive evaluations. Industrially, it supports quality prioritization, raw material selection, and origin traceability—critical for pharmaceutical compliance. Consistent with empirical evidence, this approach ensures safety and quality in *P. sibiricum*-based products by integrating multi-dimensional factors.

In summary, this research establishes a morphology-metabolism linkage paradigm, advancing TCM quality control. Future studies should dissect biosynthetic pathways underlying regional traits to optimize cultivation, ensuring clinical consistency and enhancing TCM product quality.

## 5 Conclusion

We demonstrated that high-quality *P. sibiricum* slices are typically distinguished by their elongated, yellowish-white appearance, and contain elevated levels of polysaccharides, baicalein, and 3′-methoxydaidzin. Manufacturers should concentrate on managing the five quality-determining factors identified above, while sellers and consumers can perform preliminary selection based on the length and color attributes of the slices. Our group intends to further investigate the correlation between the regional characteristics of *P. sibiricum* slices and their appearance, chemical composition, and pharmacological activities. This will lay the groundwork for the development of a quality control system for botanical drugs that embody the three excellences of shape, quality, and superior effect.

## Data Availability

The original contributions presented in the study are included in the article/supplementary material, further inquiries can be directed to the corresponding author.
